# A cross-tissue transcriptome-wide association study reveals novel susceptibility genes for migraine

**DOI:** 10.1186/s10194-024-01802-6

**Published:** 2024-06-05

**Authors:** Jianxiong Gui, Xiaoyue Yang, Chen Tan, Lingman Wang, Linxue Meng, Ziyao Han, Jie Liu, Li Jiang

**Affiliations:** Department of Neurology, National Clinical Research Center for Child Health and Disorders, Ministry of Education Key Laboratory of Child Development and Disorders, Chongqing Key Laboratory of Child Neurodevelopment and Cognitive Disorders, Children’s Hospital of Chongqing Medical University, No. 136, Zhongshan Er Road, Yuzhong District, Chongqing, 400014 China

**Keywords:** Migraine, Cross-tissue TWAS, UTMOST, Colocalization, Mendelian randomization

## Abstract

**Background:**

Migraine is a common neurological disorder with a strong genetic component. Despite the identification of over 100 loci associated with migraine susceptibility through genome-wide association studies (GWAS), the underlying causative genes and biological mechanisms remain predominantly elusive.

**Methods:**

The FinnGen R10 dataset, consisting of 333,711 subjects (20,908 cases and 312,803 controls), was utilized in conjunction with the Genotype-Tissue Expression Project (GTEx) v8 EQTls files to conduct cross-tissue transcriptome association studies (TWAS). Functional Summary-based Imputation (FUSION) was employed to validate these findings in single tissues. Additionally, candidate susceptibility genes were screened using Gene Analysis combined with Multi-marker Analysis of Genomic Annotation (MAGMA). Subsequent Mendelian randomization (MR) and colocalization analyses were conducted. Furthermore, GeneMANIA analysis was employed to enhance our understanding of the functional implications of these susceptibility genes.

**Results:**

We identified a total of 19 susceptibility genes associated with migraine in the cross-tissue TWAS analysis. Two novel susceptibility genes, *REV1* and *SREBF2*, were validated through both single tissue TWAS and MAGMA analysis. Mendelian randomization and colocalization analyses further confirmed these findings. *REV1* may reduce the migraine risk by regulating DNA damage repair, while *SREBF2* may increase the risk of migraine by regulating cholesterol metabolism.

**Conclusion:**

Our study identified two novel genes whose predicted expression was associated with the risk of migraine, providing new insights into the genetic framework of migraine.

**Supplementary Information:**

The online version contains supplementary material available at 10.1186/s10194-024-01802-6.

## Introduction

Migraine is a prevalent chronic episodic neurological disorder characterized by recurrent attacks, resulting in significant health burden, reduced quality of life, and impaired productivity [1]. It has been recognized as one of the foremost global public health concerns. The Global Burden of Disease Study 2019 (GBD2019) in *Lancet* revealed that migraine ranked second among all human diseases in terms of years lived with disability and was the leading cause of disability-adjusted life years in females aged 15–49 [2], exerting substantial adverse impacts on patients, their families, and society at large. Globally, approximately 1.04 billion individuals suffer from migraine, with a lifetime prevalence estimated at around 8.6% for males and 17% for females [3, 4].

Based on twin and family studies, the heritability of migraine ranges from 35 to 60%, indicating the significant contribution of genetic variation to the susceptibility of migraine [5, 6]. The occurrence of migraine is primarily attributed to a polygenic nature, wherein multiple genetic variants with modest individual effects collectively contribute to the development of the disorder, despite the fact that a single genetic mutation can be sufficient to trigger specific types of migraines such as familial hemiplegic migraine and migraine with aura associated with hereditary small-vessel disorders [7]. In recent years, a genome-wide association study (GWAS) of migraine has identified 123 risk loci associated with the condition [8]. However, many disease trait loci identified by GWAS are situated in non-coding regions, posing challenges in assessing their functional significance [9]. Additionally, complex linkage disequilibrium (LD) can obscure the identification of causal variants driving these associations [10].

Transcriptome-wide association studies (TWAS) integrate expression quantitative trait loci (eQTL) with summary statistics from GWAS to precisely identify candidate genes and investigate gene-trait associations [11]. However, a cross-tissue TWAS methodology known as Unified Test for Molecular Signature (UTMOST) performs gene-level association analyses across multiple tissues [12]. In contrast to single-tissue approaches, this method improves the accuracy and efficacy of imputation models by applying a “group-lasso penalty” that promotes the discovery of shared eQTL effects across different tissues while preserving robust tissue-specific eQTL effects. In recent years, cross-tissue association analysis has been extensively employed in the identification of candidate susceptibility genes for complex multisystem disorders such as rheumatoid arthritis [13], lung cancer [14], and autism spectrum disorder [15]. The present study has unveiled that aside from the prominent role of neurovascular units in migraine [7], there exist interconnections with other tissue units [16, 17].

In this study, we conducted cross-tissue TWAS analysis by integrating migraine GWAS data from the FinnGen R10 with eQTL files from the Genotype-Tissue Expression Project (GTEx) v8. Functional Summary-based Imputation (FUSION) was employed for the assessment of associations in each tissue [18], and Multi-marker Analysis of Genomic Annotation (MAGMA) was performed for validation [19]. Mendelian randomization (MR) and colocalization analysis were carried out on candidate genes, followed by subsequent bioinformatics analyses to explore their biological properties.

## Materials and methods

**Fig. 1:**
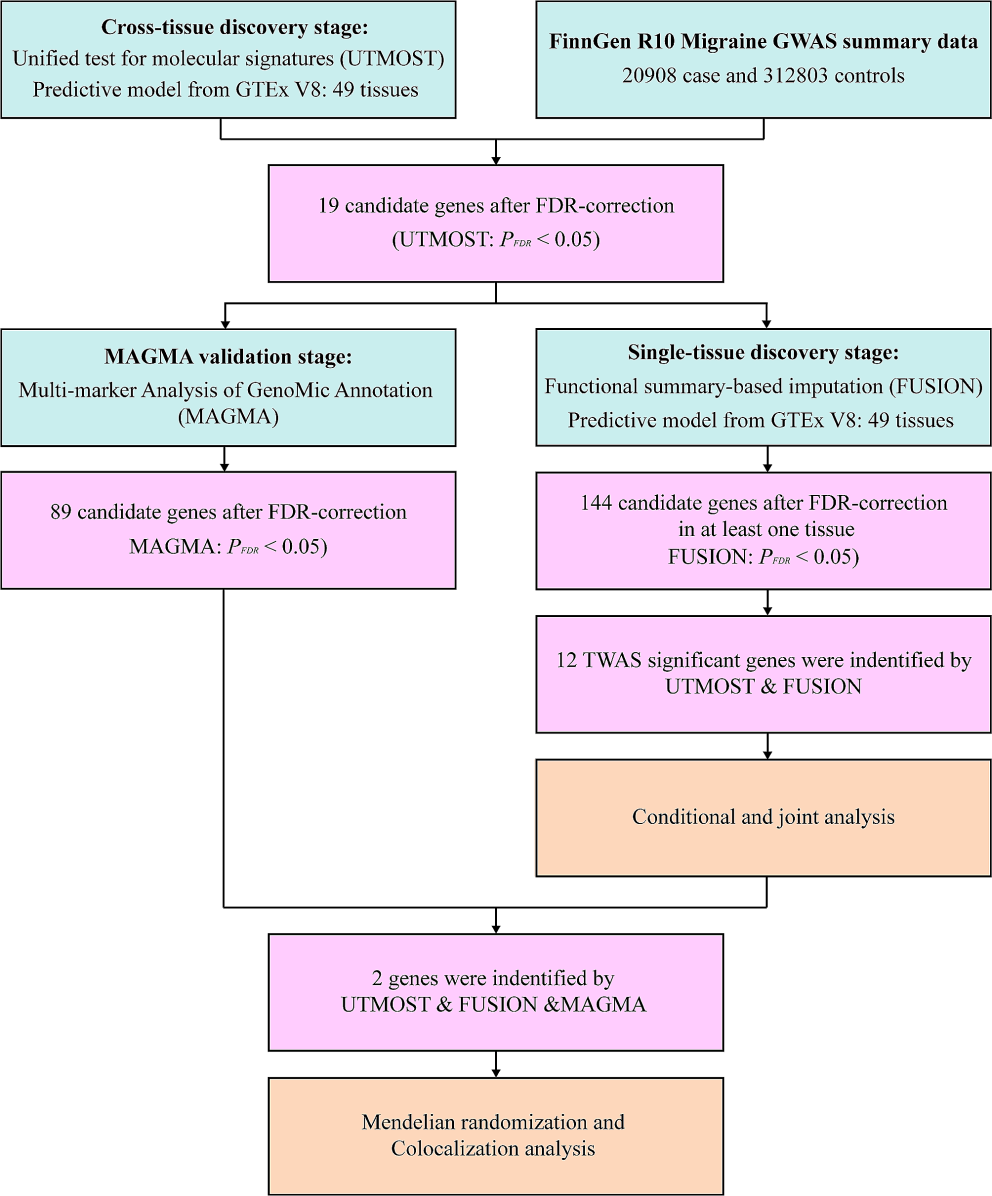
The flowchart of this study. GWAS, genome-wide association; GTEx, Genotype-Tissues Expression Project; TWAS, transcriptome-wide association studies; UTMOST, unified test for molecular signatures; FUSION, functional summary-based imputation; MAGMA, multi-marker Analysis of GenoMic Annotation

The analysis process is illustrated in Fig. 1.

### Migraine GWAS data source

The migraine GWAS data were obtained from the FinnGen R10 dataset (https://www.finngen.fi/en), which consisted of 20,908 cases and 312,803 controls of European ancestry.

### eQTL files source

The GTEx V8 dataset [20] encompasses a wealth of gene expression data from 49 different tissues, collected from 838 post-mortem donors (https://ftp.ebi.ac.uk/pub/databases/spot/eQTL/imported/GTEx_V8). The sample sizes varied across different tissues, ranging from 73 samples in the renal cortex to 706 samples in the skeletal muscle.

### TWAS analyses in cross-tissue

We employed UTMOST analyses (https://github.com/Joker-Jerome/UTMOST?tab=readme-ov-file) in cross-tissue to quantify the overall gene-trait associations at the organismal level. This approach enabled the identification of a greater number of genes within tissues with enriched trait heritability and enhanced imputation accuracy [12, 13]. Subsequently, we employed the generalized Berk-Jones (GBJ) test to integrate gene-trait associations by utilizing covariance from single-tissue statistics [12, 21]. After applying the false discovery rate (FDR) correction, a significance level of FDR < 0.05 was considered statistically significant.

### TWAS analyses in single tissue

We employed the FUSION tool (http://gusevlab.org/projects/fusion/) to conduct TWAS integrating migraine GWAS data with eQTL data from GTEx V8 49 tissues to estimate the association of each gene to disease [22]. Initially, the LD between the prediction model and the SNP at each locus of GWAS was estimated using 1,000 Genomes European samples. Subsequently, FUSION integrates several predictive models (BLUP, BSLMM, LASSO, Elastic Net, and top 1) to evaluate the overall impact of SNPs on gene expression weights. The model demonstrating the highest predictive performance was then utilized for determining the gene weights [23]. Following this, we combined the genetic effect of migraine (migraine GWAS Z-score) with these gene weights to conduct the TWAS of migraine. The subsequent study included candidate genes that met the following two criteria: (1) FDR < 0.05 in cross-tissue TWAS analysis; and (2) FDR < 0.05 in at least one tissue in single-tissue TWAS analysis.

### Conditional and joint analysis

In FUSION, we may identify multiple associated features within a locus and aim to determine which of these are conditionally independent. Therefore, we conducted conditional and joint (COJO) analysis (the post-process module in FUSION) to identify independent genetic signals [22]. The COJO analysis ensures a more comprehensive understanding of the genetic architecture underlying trait variation by accounting for LD between markers [24]. Following testing, genes that represent independent associations were referred to as jointly significant, while those that no longer showed significance were considered marginally significant.

### Gene analysis

For gene analysis, we utilized MAGMA software (version 1.08) with default parameters to aggregate SNP-level association statistics into gene scores, enabling the quantification of each gene’s degree of association with the phenotype [25, 26]. For detailed information regarding parameter settings and comprehensive methodological justifications, please refer to the original MAGMA documentation [19].

### Mendelian randomization and bayesian colocalization

We conducted MR analysis using the “TwoSampleMR” R package [27]. In this process, we utilized cis-eQTL SNPs as instrumental variables (IVs), gene expression as the exposure, and migraine GWAS as the outcome, respectively. Initially, we selected genome-wide significant SNPs (*p* < 5E-08) and performed LD clumping to obtain independent SNPs (r^2^ < 0.001) [18]. As only one standalone IV was available, we estimated the MR effect using the Wald ratio with a significance level set at *p* < 0.05.

Subsequently, we conducted a Bayesian colocalization analysis using the “coloc” R package [14, 28] to ascertain whether there is overlap between GWAS and eQTL signals in terms of causal variation loci. This analysis emphasizes the posterior probability (PP) of five relationships [28], with our belief that PP.H4 > 0.75, indicating shared causal variants between GWAS and eQTL [14, 29].

### GeneMANIA analysis

The GeneMANIA platform [30] (https://genemania.org/) integrates diverse genetic interaction, pathway, and co-expression datasets for target genes, along with other gene-function relationships, to enhance comprehension of the underlying biological functions of these targets [31].

## Results

### TWAS analyses in cross-tissue and single tissue

In the cross-tissue TWAS analysis, a total of 272 genes with *p* < 0.05 were found, (Table S1), out of which 19 genes remained significant even after FDR correction (FDR < 0.05) (Table 1). For the validation of the single-tissue TWAS analysis, a total of 144 genes with FDR < 0.05 in at least one tissue were identified (Table S2). The statistical outcomes of the 19 genes identified through cross-tissue TWAS analysis are illustrated in Figure S1. A total of 12 candidate genes met strict thresholds in both cross-tissue and single-tissue analyses, comprising 9 coding protein genes (*TEF*, *SREBF2*, *LYG2*, *XRCC6*, *CCDC134*, *SNU13*, *REV1*, *EP300*, and *LYG1*) and 3 non-coding protein genes (*RP5-821D11.7*, *AC109826.1*, and *LINC00634*) (Table S3).

**Table 1:** The significant genes for migraine risk in cross-tissue UTMOST analysis

Gene symbole	CHR	Ensemeble ID	Location (hg38)	Test score	*p* value	FDR
TEF	22	ENSG00000167074	41,367,333 − 41,399,326	14.76	1.04E-07	3.88E-04
DNPEP	2	ENSG00000123992	219,373,546 − 219,400,022	13.41	5.82E-07	1.09E-03
MEI1	22	ENSG00000167077	41,699,499 − 41,799,456	10.94	6.31E-06	5.91E-03
MKL1	22	ENSG00000196588	40,410,290 − 40,636,702	11.92	5.26E-06	5.91E-03
RP5-821D11.7	22	ENSG00000184068	41,831,215 − 41,834,665	11.17	9.44E-06	7.06E-03
AC109826.1	2	ENSG00000226791	98,761,938 − 98,772,920	11.68	1.42E-05	8.83E-03
SREBF2	22	ENSG00000198911	41,866,831 − 41,907,308	10.72	1.82E-05	8.90E-03
LINC00634	22	ENSG00000205704	41,952,165 − 41,958,933	10.06	1.90E-05	8.90E-03
SEPT3	22	ENSG00000100167	41,976,272 − 41,998,221	10.33	3.12E-05	1.17E-02
L3MBTL2	22	ENSG00000100395	41,205,205 − 41,231,271	9.54	3.13E-05	1.17E-02
LYG2	2	ENSG00000185674	99,242,246 − 99,255,282	9.31	3.97E-05	1.35E-02
TOB2	22	ENSG00000183864	41,433,492 − 41,447,023	9.77	5.68E-05	1.64E-02
XRCC6	22	ENSG00000196419	41,621,119 − 41,664,048	8.94	5.63E-05	1.64E-02
CCDC134	22	ENSG00000100147	41,800,679 − 41,826,299	8.52	8.86E-05	2.37E-02
LIPT1	2	ENSG00000144182	99,154,965 − 99,163,157	7.96	1.13E-04	2.82E-02
SNU13	22	ENSG00000100138	41,673,930 − 41,690,504	8.49	1.68E-04	3.70E-02
REV1	2	ENSG00000135945	99,401,327 − 99,490,035	8.88	1.66E-04	3.70E-02
EP300	22	ENSG00000100393	41,091,786 − 41,180,079	7.98	2.06E-04	4.14E-02
LYG1	2	ENSG00000144214	99,284,238 − 99,304,742	7.05	2.10E-04	4.14E-02

### COJO analysis

The 12 candidate genes, primarily located on chromosomes 2 and 22, underwent COJO analysis in their respective tissues to eliminate false positive results arising from LD (Table S4). In Cells_Cultured_fibroblasts, conditioning on the predicted expression of *REV1* led to a notable reduction in the TWAS signal for *LYG1* (Figure S2A). In Colon_Transverse, the TWAS signal for *LYG2* significantly decreased when conditioned on the predicted expression of *LYG1* (Figure S2B). In Lung, conditioning on the predicted expression of *RP5-821D11.7* resulted in a substantial drop in the TWAS signals for *XRCC6* and *CCDC134* (Figure S2C). Moreover, in Skin_Sun_Exposed_Lower_leg the TWAS signals for *RP5-821D11.7*, *CCDC134*, and TEF markedly diminished when conditioned on the predicted expression of *SREBF2* (Figure S2D). Similarly, in Whole_Blood, the TWAS signal for *LYG1* showed a significant decline when the analysis was conditioned on the predicted expression of *REV1* (Figure S2E). Due to *TEF*, *LYG2*, and *XRCC6* achieving significance solely in the TWAS results for an individual tissue and the potential influence of LD on these findings, these genes were not included in further analyses.

### Gene analysis of MAGMA

**Fig. 2:**
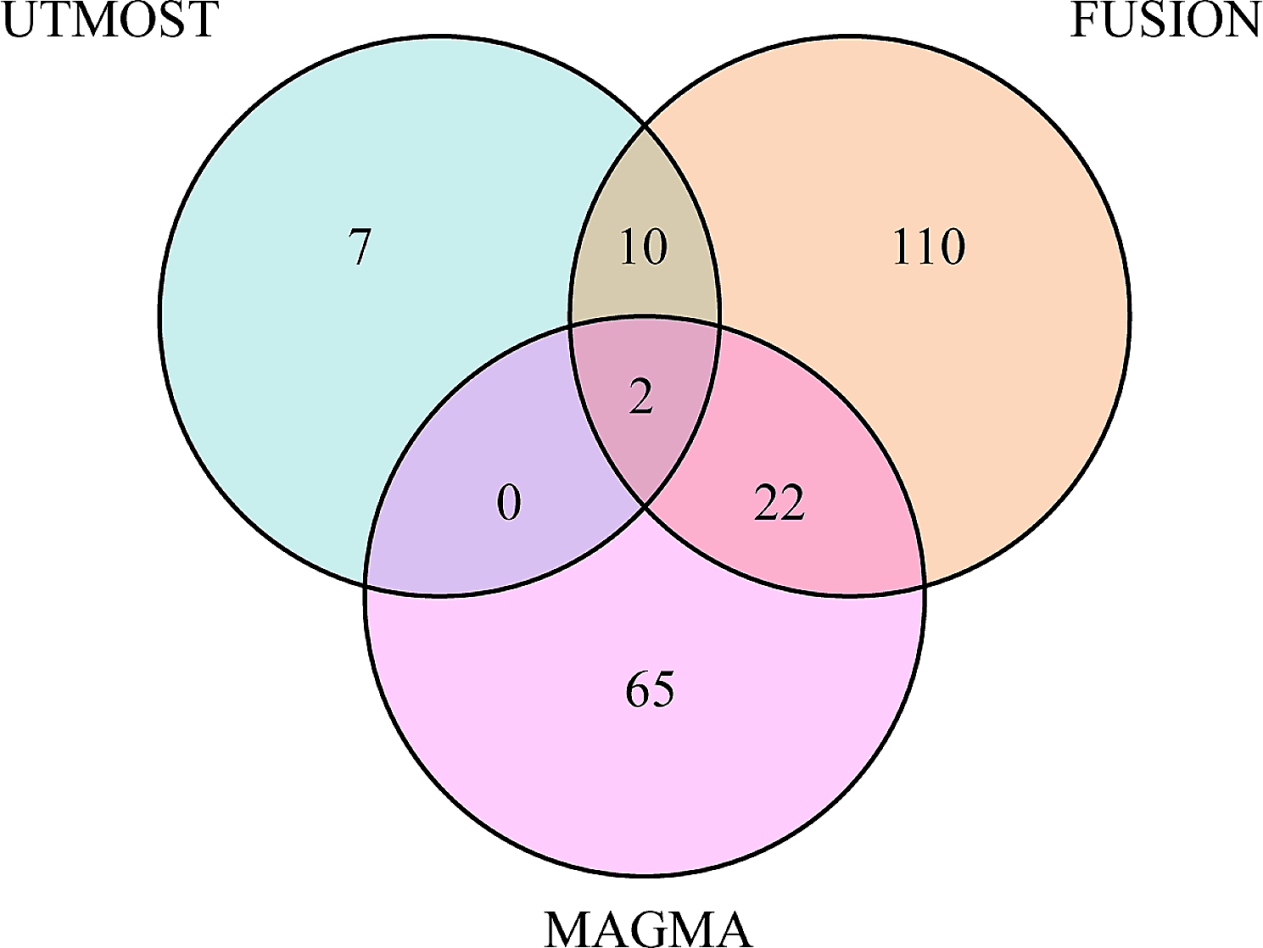
Venn diagram. MAGMA identified 89 significant genes associated with hypertension, FUSION identified 144, and UTMOST cross-tissue analysis identified 19, of which 2 were common

MAGMA gene-based test identified 89 significant genes associated with migraine (FDR < 0.05) ((Figure S3 and Table S5). To enhance the robustness of our findings, we integrated the UTMOST cross-tissue results with the significant genes detected by FUSION and MAGMA, resulting in the identification of two promising candidate genes (*REV1* and *SREBF2*) (Fig. 2).

### MR and colocalization results

**Fig. 3:**
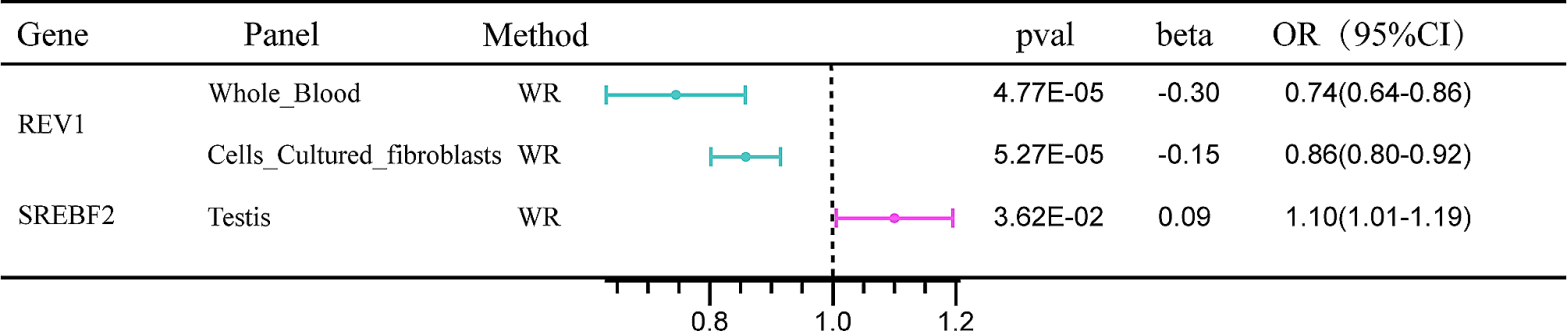
The results of colocalization analysis between candidate genes and migraine. The SNP rs17022564 exhibited the lowest cumulative sum of migraine GWAS and *REV1* eQTL p values both in Whole_Blood *(A)* and Cells_Cultured_fibroblasts *(B)*. The SNP rs738248 exhibited the lowest cumulative sum of migraine GWAS and *SREBF2* eQTL p values both in Skin_Sun_Exposed_Lower_leg *(C)* and Testis *(D)*

**Fig. 4:**
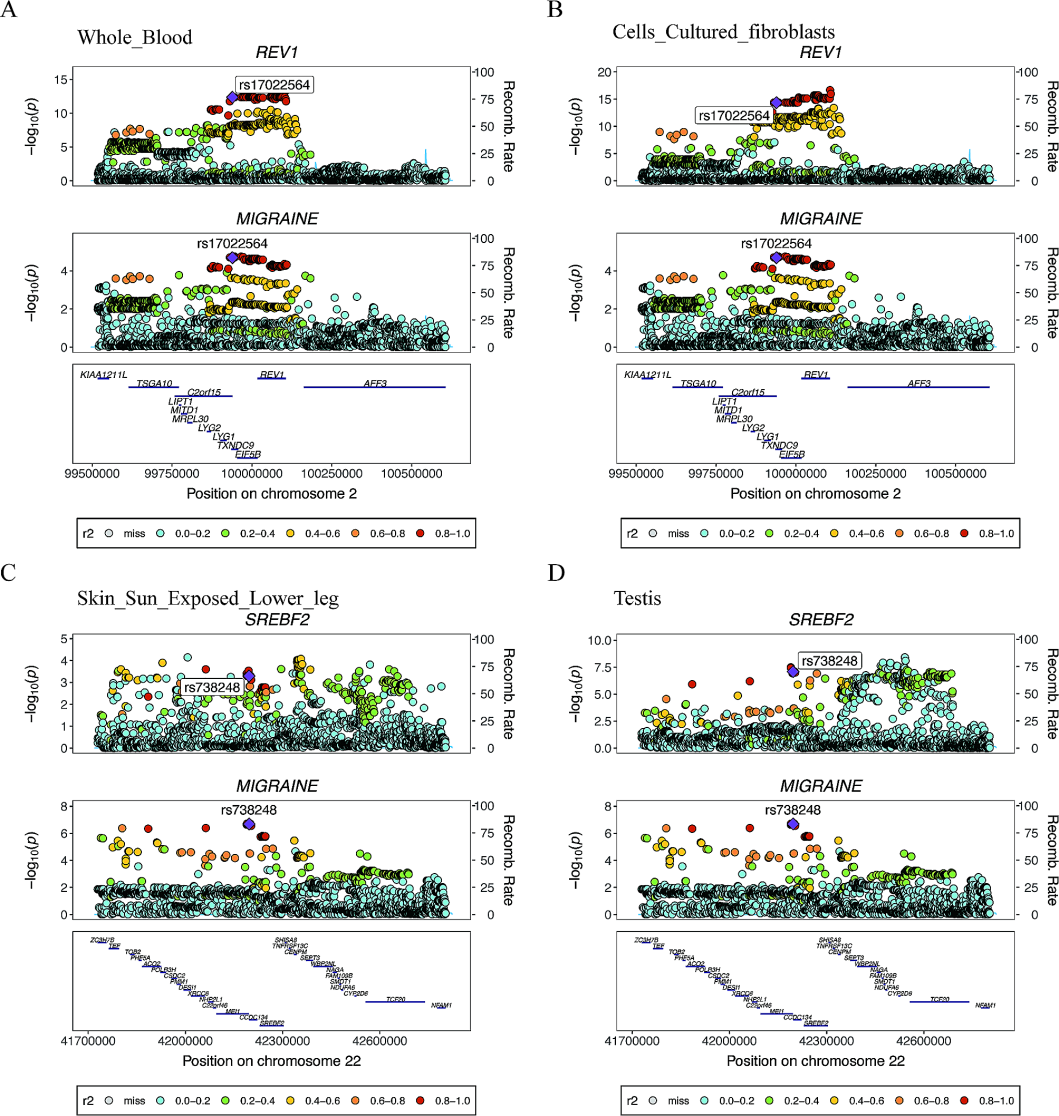
The MR results confirmed the causal associations between two candidate genes and migraine

The *REV1* gene is located on chromosome 2q11.2, and FUSION analysis revealed its significant association with migraine in Whole_Blood and Cells_Cultured_fibroblasts. MR analyses confirmed a causal relationship between *REV1* and migraine (*p* < 0.05). The odds ratios (OR) (95% confidence intervals (CI)) were estimated at 0.74 (0.64, 0.86) for Whole_Blood and 0.86 (0.80, 0.92) for Cells_Cultured_fibroblasts (Fig. 3 and Table S6). Subsequent colocalization analysis further supported this finding, with PP.H4 of 0.88 and 0.85 for Whole_Blood and Cells_Cultured_fibroblasts, respectively (Table S7). Notably, rs17022564 emerged as the most significant co-localization locus for migraine in both tissues (Fig. 4A, B).

The *SREBF2* gene is located on chromosome 22q13.2, and the FUSION results demonstrate its significant expression in Skin_Sun_Exposed_Lower_leg and Testis tissues. MR analysis of Testis tissues confirmed a significant causal association with migraine (*p* < 0.05), with an OR (95%CI) of 1.10 (1.01, 1.19). However, no eligible IVs were found in the Skin_Sun_Exposed_Lower_leg tissue (Fig. 3 and Table S6). Furthermore, Colocalization analysis revealed PP.H4 values of 0.73 and 0.87 for these tissues, respectively (Table S7). Among them, rs738248 was identified as the most significant colocalized locus with migraine in both Skin_Sun_Exposed_Lower_leg and Testis tissues (Fig. 4C, D).

### GeneMANIA analysis

**Fig. 5:**
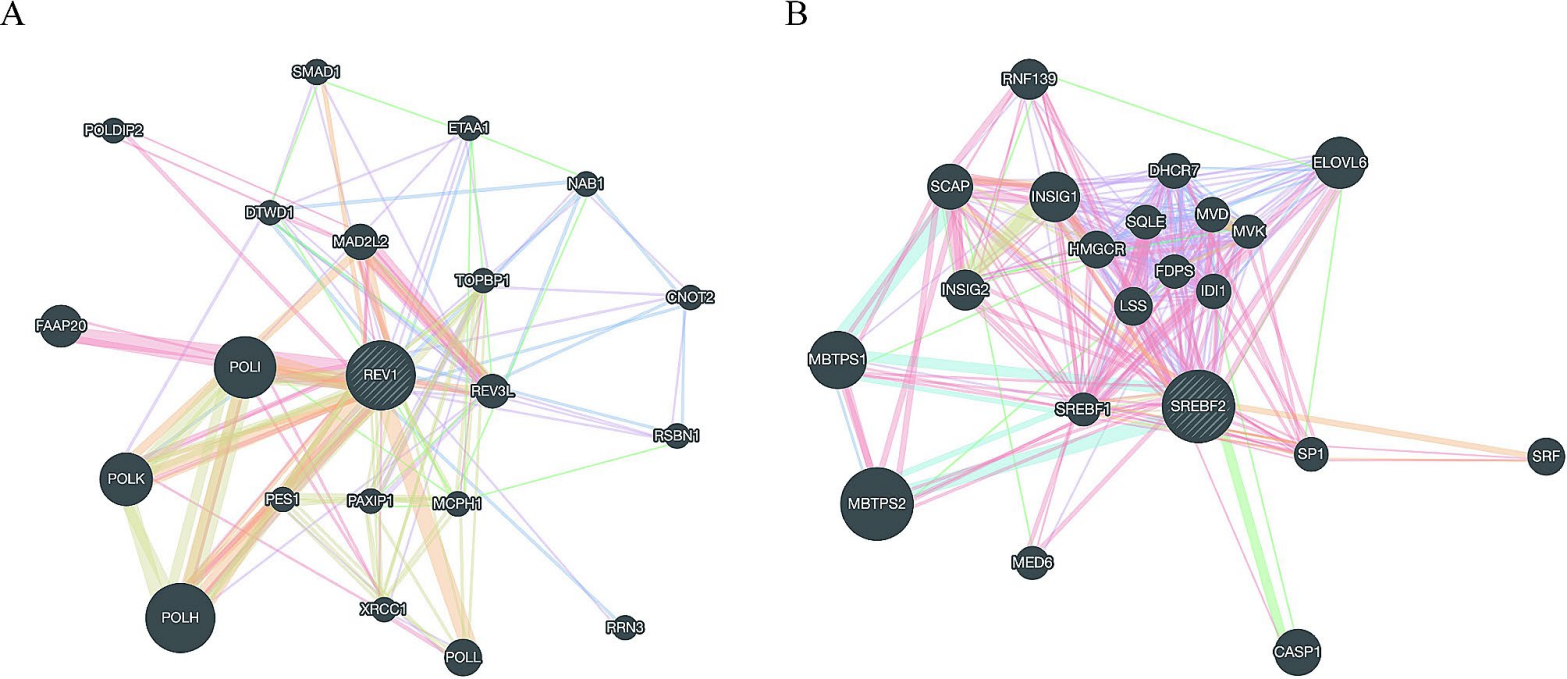
GeneMania gene network. *(A)REV1* as the core, and *(B)SREBF2* as the core

The potential interaction gene network constructed with *REV1* as the core is shown in Fig. 5A. The most significant functional pathways enriched in *REV1*-related gene networks are postreplication repair, DNA synthesis involved in DNA repair, and translesion synthesis (Table S8). The constructed gene interaction network, with *SREBF2* at its nexus, is depicted in Fig. 5B. The most significant functional pathways enriched in *SREBF2*-related gene networks are sterol biosynthetic process, secondary alcohol biosynthetic process, and cholesterol metabolic process (Table S9).

## Discussion

Utilizing migraine GWAS and GTEx V8 eQTL data, we systematically evaluated the relationship between genetic predisposition for gene expression and the risk of migraine. Cross-tissue TWAS analysis, along with validation through single-tissue TWAS and MAGMA, led to the identification of two migraine susceptibility genes (*REV1* and *SREBF2)*, which were further substantiated by MR and colocalization analyses. Bioinformatic analyses enhanced our understanding of the potential functions of these susceptibility genes.

The utilization of multi-omics association studies is currently prevalent in the identification of disease susceptibility genes. The most extensive GWAS meta-analysis of migraine to date has identified 73 potential genes associated with susceptibility to migraine through fine-mapping of causal gene-sets and TWAS analysis [8]. Li et al. identified five genes associated with migraine in human brain tissue and plasma proteomic and transcriptomic analyses, which are mainly expressed in ependymal cells, neurons, and glial cells [23]. Another study utilized imputation models of three TWAS, MASHR, elastic net, and SMultiXcan to identify risk loci for migraine GWAS and potential susceptibility genes [32]. Meyers et al. also employed a TWAS analysis, utilizing eQTL data from GTEx V7, to identify novel candidate susceptibility genes associated with migraine [33]. The variation in outcomes may be attributed to disparities in sample size and source, as well as discrepancies among algorithms employed. However, this emphasizes the significance of conducting multiple avenues of research to identify potential genetic factors contributing to migraine susceptibility. To a certain extent, both of these studies incorporated cross-tissue TWAS analysis [32, 33]; however, it is imperative to further validate the outcomes of the cross-tissue analysis, as their primary focus remained on investigating the association between genes in single tissue and migraine. Moreover, in comparison to GTEx V7, the updated version V8 encompasses a 49% increase in RNA-seq samples derived from 33% more tissue donors, alongside the inclusion of splicing eQTLs [34]. In this study, the cross-tissue TWAS analysis of UTMOST was employed as the core, which improves upon single-tissue TWAS by integrating gene expression data from multiple tissues, increasing statistical power to identify genes associated with complex traits, providing a more comprehensive understanding of gene-trait associations, and enhancing the ability to detect missed associations when analyzing a single tissue in isolation [12]. Currently, the analytical approach of cross-tissue TWAS identification and single-tissue TWAS and gene analysis verification, combined with colocalization and MR methodology has been employed in numerous studies to identify susceptible genes associated with various diseases [13, 14, 29]. Through cross-tissue TWAS analysis and rigorous validation, two genes (*REV1* and *SREBF2*) associated with migraine risk were identified, which have not been previously reported.

*REV1* is a member of the translesion synthesis DNA polymerase Y family and exhibits widespread expression in various tissues throughout the body. It plays an indispensable role in diverse DNA replication activities, performing crucial functions in both spontaneous and DNA damage-induced mutagenesis [35]. The diseases associated with REV1 encompass the variant form of xeroderma pigmentosum [36] and Fanconi anemia [37]. In this study, the MR results indicated a significant causal association between *REV1* expression and a reduced risk of migraine. Although there was no prior empirical evidence linking *REV1* to migraine, we extrapolated conclusions from its functional characteristics. In *REV1* knockout mice, endogenous DNA damage caused DNA replication stress [38]. Plasma levels of 8-OHdG, a marker for DNA damage, were significantly elevated in migraine patients compared to controls [39]. Moreover, the frequency of headache attacks showed a significant correlation with weather variables, such as the UV index and duration of sunshine [40]. Furthermore, *REV1* has been demonstrated to participate in the DNA damage tolerance induced by UV irradiation in mammals. The expression of *REV1* alone was sufficient to augment the tolerance to UV-induced DNA damage [41]. The inverse correlation between *REV1* expression and migraine risk may be attributed to its regulated role in DNA damage repair, although further studies are required for confirmation.

The *SREBF2* gene encodes a transcription factor that is widely expressed and plays a crucial role in maintaining cholesterol homeostasis by regulating the transcription of sterol-regulated genes. Diseases associated with *SREBF2* include atherosclerosis [42] and adrenoleukodystrophy [43]. The MR analysis revealed a significant association between *SREBF2* expression and the susceptibility to elevated migraine risk. The *SREBF2* gene mutations were identified in patients with autosomal dominant hypercholesterolemia, which may be correlated with elevated levels of cholesterol and glucose [44]. The investigators discovered that elevated levels of lipopolysaccharide (LPS) in patients with cirrhosis resulted in dysregulation of *SREBF2* expression. Increased expression of *SREBF2* induced endoplasmic reticulum stress by elevating intracellular cholesterol levels and promoting Bax expression, thereby causing additional damage to LPS-induced endothelial cells [45]. Additionally, *SREBF2* facilitated the upregulation of *STARD4* by directly binding to its promoter region, thereby inducing elevated levels of mitochondrial cholesterol, and contributing to the resistance of hepatocellular carcinoma against sorafenib [46]. It has been suggested that migraine frequency and intensity were significantly positively correlated with serum cholesterol levels [47]. In another cross-sectional study, an association was observed between elevated levels of total cholesterol and triglycerides and the occurrence of migraine with aura in older adults [48]. Large-scale plasma metabolomics studies found a consistent association between migraine and decreased high-density lipoprotein (HDL) levels [49]. Considering the significant role of SREBF2 in regulating cholesterol metabolism and the strong correlation between lipid metabolism and migraine, we postulated that the positive association between SREBF2 expression and migraine risk may be attributed to its involvement in cholesterol metabolism regulation.

With the large-scale GWAS, numerous reliable SNPs and genes associated with genetic risk factors for migraine have been identified [8, 50]. However, there exist evident challenges in establishing connections between relevant SNPs and genes with potential pathophysiological pathways. Current hypotheses propose that vascular function, metal ion homeostasis, neuronal function, and ion channel activity may be implicated in the pathophysiology of migraine; nevertheless, these notions still necessitate validation through high-throughput experiments conducted on cellular and animal models [51]. We sought to explore the potential interactions among SREBF2 or REV1 and previously identified genes associated with migraine susceptibility [8, 23, 32, 33, 50–53]. However, substantial evidence is currently lacking. Therefore, further investigation is required to elucidate the role of SREBF2 and REV1 in migraine pathogenesis.

In summary, the present study identified two novel genes associated with migraine susceptibility, and their potential functions were speculated based on the available results. Our study had several limitations worth noting. Firstly, our sample was exclusively European, limiting the generalizability of our findings across diverse populations. Secondly, despite our multi-faceted approach to reduce the incidence of false positives, the absence of an independent replication dataset posed a limitation to the validation of our results. Lastly, the expression levels of *REV1* and *SREBF2* in tissues previously more closely associated with migraine, such as cerebral arteries, were not able to be assessed and validated in the current dataset. In future studies, a series of biological experiments will be required to corroborate our hypothesized pathophysiological mechanisms. Nevertheless, this study offered novel perspectives and deductions regarding the underlying pathophysiological mechanisms of migraine.

## Conclusion

In conclusion, our cross-tissue TWAS analysis identified two novel susceptibility genes whose expression correlated with the risk of migraine, contributing fresh insights into the genetic architecture of this migraine. However, further functional research is necessary to elucidate the potential biological activity of these significant signals.

### Electronic supplementary material

Below is the link to the electronic supplementary material.

Supplementary Material 1

Supplementary Material 2

## Data Availability

The migraine GWAS data were obtained from the FinnGen R10 dataset (https://storage.googleapis.com/finngen-public-data-r10/summary_stats/finngen_R10_G6_MIGRAINE.gz). Gene expression and eQTL data are freely available at https://ftp.ebi.ac.uk/pub/databases/spot/eQTL/imported/GTEx_V8.
