# Supplementary material for: A cross-tissue transcriptome-wide association study reveals novel susceptibility genes for migraine
Source: J Headache Pain. 2024 Jun 5;25(1):94. doi: 10.1186/s10194-024-01802-6 (PMC11151630; doi:10.1186/s10194-024-01802-6)
Supplement: Supplementary file 2 — Supplementary Material 2 [file 10194_2024_1802_MOESM2_ESM.docx]

**A Cross-Tissue Transcriptome-Wide Association Study Reveals Novel Susceptibility Genes for Migraine**

**Supplementary Figures**

**
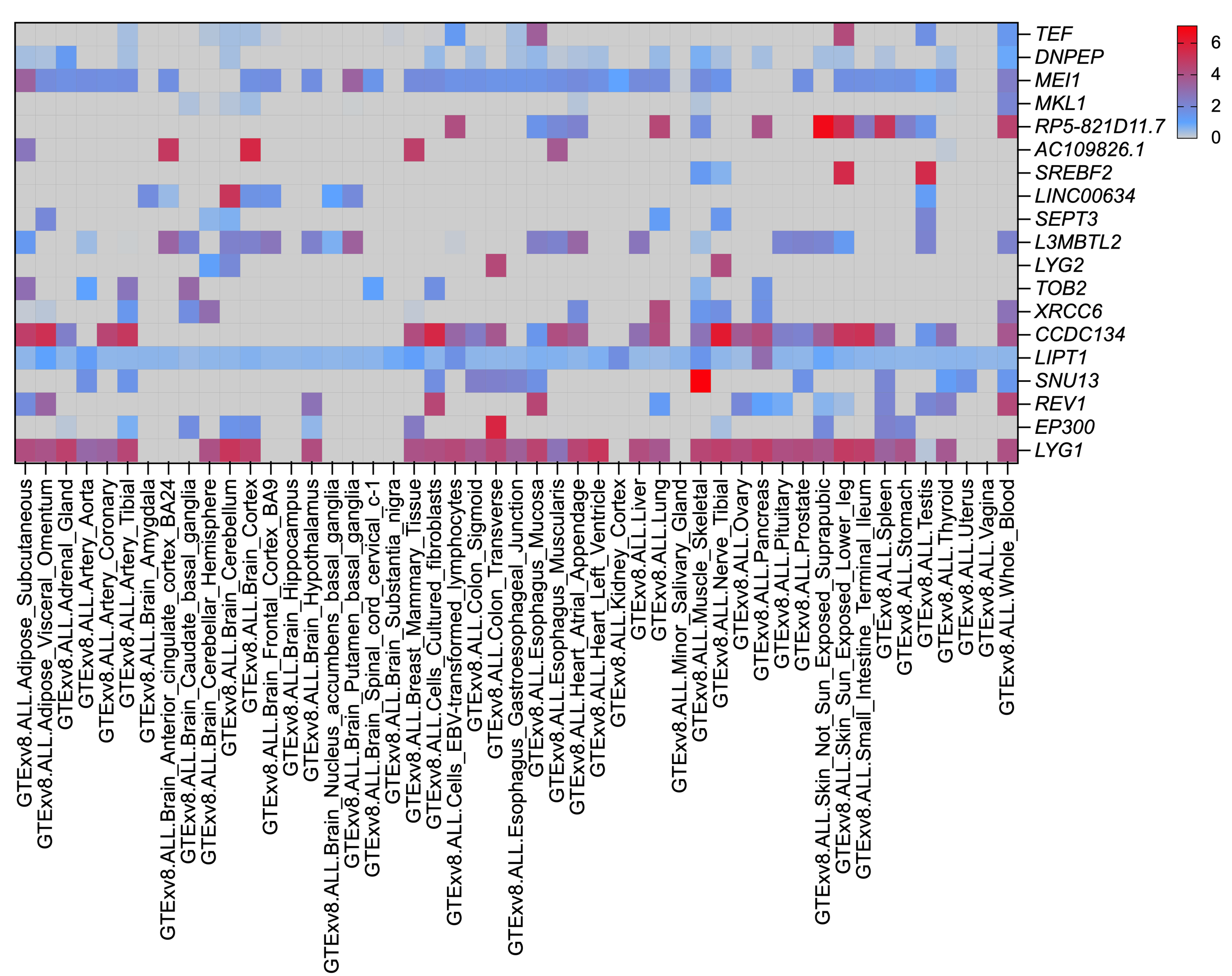
**

**Figure S1.** The heatmaps of the 19 genes split out by tissues. The statistics represent the p value of FUSION in -log10 scale. The gray lattice represents statistics not available.


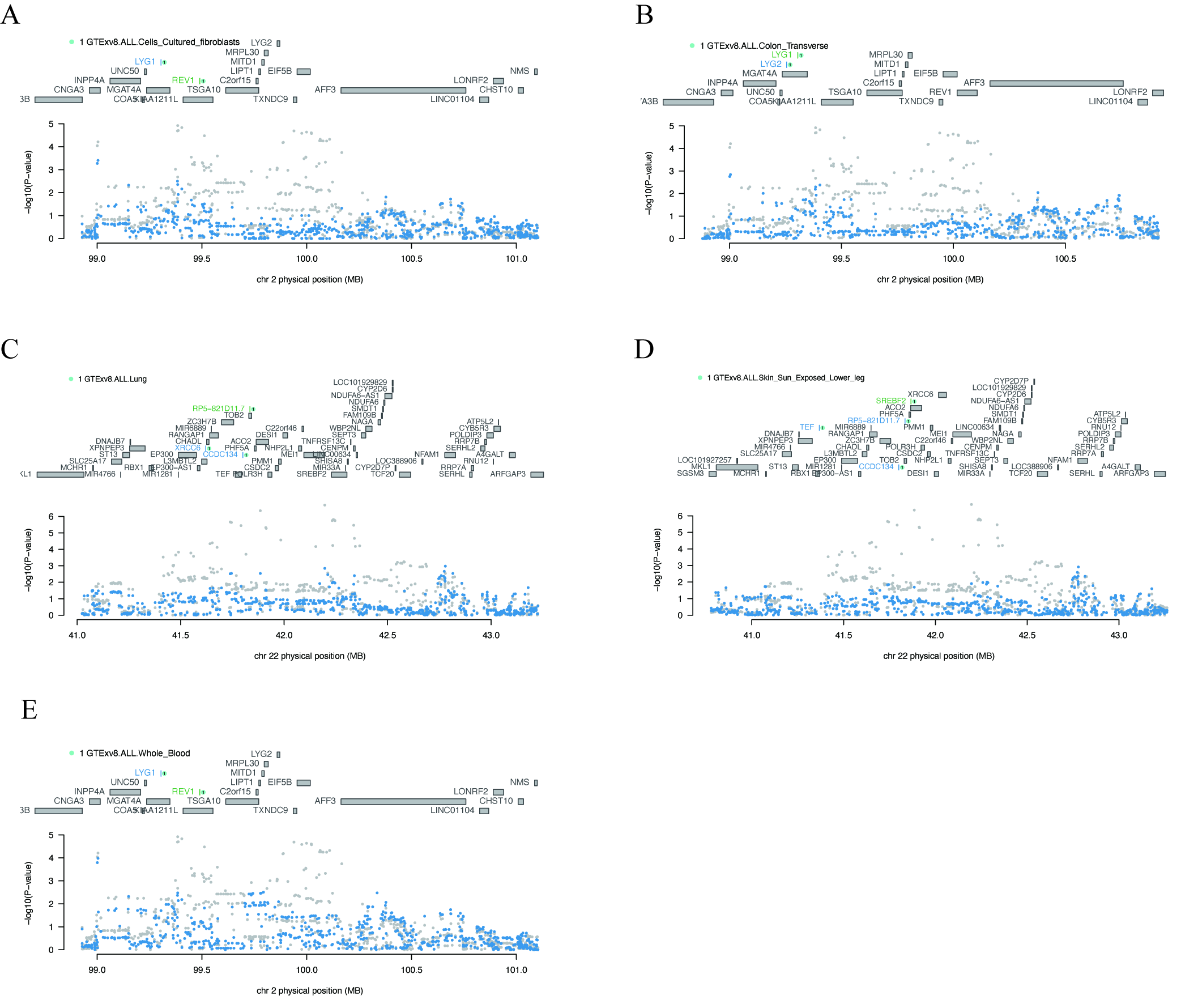


**Figure S2.** Regional association of TWAS hits. (A), (B), and (E) Chromosome 2 regional association plot. (C) and (D) Chromosome 22 regional association plot. The top panel highlights all genes in the region. The marginally associated TWAS genes are shown in blue, and the jointly significant genes are shown in green. The bottom panel shows a regional Manhattan plot of GWAS data before (grey) and after (blue) conditioning on the predicted expression of the green genes.

**Figure S3.** Manhattan plot of the MAGMA results for migraine. Each data point on the plot represents a specific gene’s association with migraine, ranked based on its genomic location along the X-axis and the strength of association (-log10(P) from the z-score test) along the Y-axis. MAGMA analysis revealed that 89 genes were significantly associated with migraine, as indicated by an FDR < 0. 05. The red horizontal line represents the significance threshold for FDR =0.05.
